# F2r negatively regulates osteoclastogenesis through inhibiting the Akt and NFκB signaling pathways

**DOI:** 10.7150/ijbs.41867

**Published:** 2020-03-12

**Authors:** Yan Zhang, He Wang, Guochun Zhu, Airong Qian, Wei Chen

**Affiliations:** 1 Laboratory for Bone Metabolism, Key Lab for Space Biosciences and Biotechnology, School of Life Sciences, Northwestern Polytechnical University, Xi'an, Shaanxi, 710072, China.; 2 Department of Pathology, The School of Medicine, University of Alabama at Birmingham, Birmingham, AL 35294, USA.

**Keywords:** F2r, osteoclasts, bone resorption, Akt signaling pathway, NFκB signaling pathway

## Abstract

G-protein-coupled receptors (GPCRs) are pivotal drug targets for many diseases. Coagulation Factor II Thrombin Receptor (F2R) is an important member of GPCR family that is highly expressed in osteoclasts. However, the role of F2r in osteoclasts is still unclear. Here, to examine the functions of F2r on osteoclast formation, differentiation, activation, survival, and acidification, we employed loss-of-function and gain-of-function approaches to study F2r using F2r-targeted short hairpin RNA (sh-F2r) lentivirus and overexpression plasmid pLX304-F2r lentivirus respectively, in mouse bone marrow cells (MBMs) induced osteoclasts. We used three shRNAs targeting F2r which had the ability to efficiently and consistently knock down the expression of F2r at different levels. Notably, F2r knockdown trigged a significant increase in osteoclast activity, number, and size, as well as promoted bone resorption and F-actin ring formation with increased osteoclast marker gene expression. Moreover, F2r overexpression blocked osteoclast formation, maturation, and acidification, indicating that F2r negatively regulates osteoclast formation and function. Furthermore, we investigated the mechanism(s) underlying the role of F2r in osteoclasts. We detected RANKL-induced signaling pathways related protein changes F2r knockdown cells and found significantly increased pAkt levels in sh-F2r infected cells, as well as significantly enhanced phosphorylation of p65 and IKBα in early stages of RANKL stimulation. These data demonstrated that F2r responds to RANKL stimulation to attenuate osteoclastogenesis through inhibiting the both F2r-Akt and F2r-NFκB signaling pathways, which lead a reduction in the expression of osteoclast genes. Our study suggests that targeting F2r may be a novel therapeutic approach for bone diseases, such as osteoporosis.

## Introduction

Osteoclasts are multinucleated cells derived from bone marrow monocyte/macrophage precursors 1,2. Osteoclast differentiation from osteoclast precursors - mouse bone marrow cells (MBMs) is stimulated by two key cytokines: macrophage colony-stimulating factor (M-CSF) and receptor activator of nuclear factor-κB ligand (RANKL) 1,3. Osteoclasts play a vital role in bone remodeling to maintain bone structure and function. Excessive osteoclastic activity causes an imbalance in bone remodeling, which leads to various bone diseases including osteoporosis, periodontal disease, rheumatoid arthritis, multiple myeloma and metastatic cancers 2,4,5. Thus, targeting negative regulators of osteoclasts may serve as an effective approach to treat pathological bone diseases related to osteoclast over-activation 6. However, negative osteoclast regulators have received limited attention and warrant further study. G-protein-coupled receptors (GPCR) located in the cell membrane that have evolved to couple extracellular signals into intracellular responses, and have been identified as effective targets for identifying novel therapeutic agents 7-10. Twenty to fifty percent of FDA-approved drugs act on GPCRs in some way 11-14. A few GPCRs have been reported to regulate osteoclast and bone related diseases 15,16, but there are more functions of GPCRs on osteoclast needed to be elucidated.

In our study, we detected the different expression of GPCRs in monocytes and osteoclast by RNA-seq. Notably, Coagulation Factor II Thrombin Receptor (F2R) was highly expressed in osteoclasts. F2r is an important member of the G-protein-coupled receptor (GPCR) family which is also known as Proteinase-Activated Receptor 1 (PAR1) 17,18. F2r is activated by thrombin or another proteinase, causing diverse biologic activities. It is involved the progression and metastasis of various types of tumors, including sarcoma, melanoma, breast cancer, lung cancer, prostate cancer and pancreatic cancer 17-20. F2r also plays an important role in coagulation and inflammation in response to injury 18, 21-23. However, the effects of F2r in osteoclasts are still not clear. Here, we examined the functions of F2r in osteoclast formation, differentiation, activation, survival, and acidification by silencing and overexpressing F2r in osteoclasts precursors using lentivirus.

We also investigated the mechanism underlying the role of F2r in osteoclasts. The activation of different downstream signaling pathways (such as Akt/Gsk3β, NF-κB, ERK1/2 and p38 MAPK signaling pathways) by answering RANKL stimulation result in the expression of genes that is crucially essential to promote osteoclast differentiation, which regulated transcription factors for osteoclastogenesis 1,5. We detected RANKL-induced signaling pathways related protein changes in F2r knockdown cells and found significantly increased pAkt levels in sh-F2r infected cells, as well as significantly enhanced phosphorylation of p65 and IKBα in early stages of RANKL stimulation. Collectively, these data demonstrated that F2r responds to RANKL stimulation to attenuate osteoclastogenesis through inhibiting both F2r-Akt and F2r-NFκB signaling pathways, which lead to a reduction in the expression of osteoclast genes. Our study suggests that targeting F2r may be a novel therapeutic approach for osteolytic diseases.

## Materials and Methods

### Animal Experimentation

All animal experiments were performed according to the requirements and regulations of the Institutional Animal Care and Use Committee (IACUC) at University of Alabama at Birmingham (UAB). In our study, all experiments used wild type (WT, C57BL/6) mice that were purchased from The Jackson Laboratory.

### Cell culture

Mouse bone marrow cells (MBMs) were isolated from long bones of 8-weeks old wild type mice as described previously 5,24. Then, MBMs were cultured in α-minimal essential medium (α-MEM, Invitrogen, USA) with 10% FBS (Invitrogen) containing macrophage colony-stimulating factor (M-CSF, 20 ng/ml, R&D Systems, USA). After 12 hours, cells were cultured in α-MEM (Invitrogen, USA) with 10% FBS (Invitrogen) containing 10 ng/ml receptor activator of NF-κB ligand (RANKL, Peprotech, Rocky Hill, NJ, USA) and 10 ng/ml M-CSF to further induce to osteoclasts. Cultured osteoclasts were used for the following *in vitro* studies.

### RNA-seq analysis

The cells were isolated from primary monocytes and osteoclasts to perform RNA-sequence analysis. Primary monocytes were collected from long bone of 8-weeks old wild type mice. Osteoclasts were induced by M-CSF (10ng/ml) and RANKL (10ng/ml) for 5 days. Total RNA was isolated with TRIzol reagent (Life Technologies, Inc., Grand Island, NY, USA; 15596018). RNA samples were sequenced across three batches on an Illumina HiSeq 2000 system (Illumina, San Diego, CA) to obtain 100 base-pair single-end reads per sample 25. The results were visualized interactively in the form of heat map by Heatmapper web server 26.

### Letivirus production and transduction

There are 3 TRC lentiviral mouse F2r targeted short hairpin RNA (shRNA) that was encoded by pLKO.1 vectors (sh-F2R-1: TRCN0000218176, Clone ID: NM_010169.3-397s21c1; sh-F2r-2: TRCN0000226151, Clone ID: NM_010169.3-1522s21c1; sh-F2r-3: TRCN0000226150, Clone ID: NM_010169.3-1314s21c1; Sigma) to knockdown F2r gene expression in osteoclasts. A pLKO.1 vector encoding scrambled shRNA (sh-sc) sequence and pLKO.1 GFP shRNA also purchased from Sigma as control. There also one pLX304-F2r from the CCSB-Broad ORF Lentiviral Expression Library (TransOMIC Technologies Inc) 27 to achieve F2r overexpression in osteoclasts. The lentiviruses were generated by 293T cell line as previously described 5,28. And the viral supernatant was harvested, aliquoted and stored at -80 °C for osteoclastogenesis assays later. The titer of lentivirus was assessed by qPCR Lentivirus Titration (Titer) Kit (ABM, LV900) according to the manufacturer's instructions 27. The MBMs for lentivirus infection were cultured as above. MBMs were seeded on a plate (3.8×10^5^ cells per well of 12-well plate), and stimulated with 20 ng/ml M-CSF for 1 day. At day 2, cells were stimulated with 10ng/ml M-CSF and 10ng/ml RANKL. Lentivirus (titer: 6×10^6^ IU/ml) and 8 μg/ml polybrene were mixed gently with medium and added in the wells (12-well plate) for 24 hours in the incubator. Infected cells were stained or examined on day 4 or day 5.

### Western blot analysis

We used 10% sodium dodecyl sulfate-polyacrylamide gel electrophoresis (SDS-PAGE) to perform western blot as described 24,28,29. MBMs were transfected lentivirus and induced to 5 days for western blot analysis. For the phosphorylation assay, monocytes/macrophages (derived from MBMs with 2.5 days treatment of RANKL and M-CSF) were starved for 4 hours and stimulated by 10 ng/ml RANKL for different durations of time. We performed western blot by using following antibodies: anti-F2r IgG (Thermo Fisher, PA5-94929), anti-Ctsk IgG (Santa Cruz, sc-48353), anti-GAPDH IgG (Santa Cruz, sc-25778), anti-NFATc1 IgG (Santa Cruz, sc-7294), anti-Pu.1 IgG (Santa Cruz, sc-352), anti-Tubulin IgG (Santa Cruz, sc-5274), anti-Akt IgG (Cell Signaling Technology, 4685), anti-phospho-Akt (Thr308) IgG (Cell Signaling Technology, 2965),anti-p65 (Cell Signaling Technology, 8242); anti-phospho-p65 (Cell signaling Technology, 3033), anti-IκBα IgG (Cell Signaling Technology, 4812), anti-phospho-IκBα (Ser32) IgG (Cell Signaling Technology, 2859), anti-Erk IgG (Cell Signaling Technology, 4370), anti-phospho-Erk (Thr202/Tyr204) IgG (Cell Signaling Technology, 4695). Member images were captured by Fluor-S-Multi-Imager, then results quantified by Image J software from the National Institutes of Health 30. Results were normalized by the housekeeping protein Tubulin or glyceraldehyde-3-phosphate dehydrogenase (GAPDH).

### Quantitative RT-PCR (qRT-PCR) analysis

We isolated total RNA from cultured cells with TRIzol reagent (Life Technologies, Inc., Grand Island, NY, USA; 15596018). Total RNA was reverse-transcribed to complementary DNA (cDNA) by SuperScript VILO Master Mix (Life Technologies, 11755050) 31. We used SYBR Green reagents (Life Technologies, 4385610) and StepOne Real-Time PCR System (Life Technologies, Applied Biosystems) to perform quantitative reverse transcription PCR (qRT-PCR) 32. *Gapdh* was used for internal controls. The primer sequences are shown in Table S1.

### TRAP stain

TRAP (tartrate-resistant acid phosphatase 5) stain was performed by a leukocyte acid phosphatase kit (Sigma, 387-A) to examine osteoclasts size, osteoclasts formation and activity as described 5,33.

### WGA stain assay

Wheat germ agglutinin (WGA) stain was used to evaluate bone resorption activity as described 28. We used Peroxidase-conjugated WGA-lectin (Sigma-Aldrich, L-3892) and DAB Peroxidase (horseradish peroxidase [HRP]) Substrate Kit (Vector Laboratories, SK-4100) to perform this assay.

### Cell Immunofluorescent (IF) stain and F-actin ring stain

F-actin ring formation was evaluated as previously determined 31. MBMs were cultured on bovine cortical bone slices in 24-well plates or directly cultured in wells of 96-well plates, then induced to osteoclasts by M-CSF and RANKL. Cells were fixed with 3.7% formaldehyde 15 min, washed by cold PBS 3 times, and then permeabilized with 0.2% Triton X-100 10 min. Next, the cells were blocked with 1% goat serum, and incubated with rhodamine phalloidin (Life Technologies, USA) and anti-Ctsk antibody (Santa Cruz, sc-48353) over night, and then incubated with FITC anti-mouse IgG (HþL) secondary antibody in the dark. Stained cells were observed under a fluorescence microscope. For the quantification of IF stain, we used NIH Image J to perform counts.

### Acridine orange stain

Osteoclast acid production was detected using Acridine orange (AO) stains as previously described 31. Osteoclasts were incubated in a-MEM containing 5 mg/ml of Acridine orange (Sigma) for 15 min at 37 °C, washed, and chased for 10min in fresh media without Acridine orange in the dark. We use fluorescence microscope (Leica Texas Red filter) to observe stained cell.

### Immunofluorescence (IF) stain

Immunofluorescence stain was carried out as described 34. Wilde type newborn femur sections were fixed in 4% PFA for 15 min and heat-induced antigen retrieval was performed (abcam, ab973). The sections were permeabilized with 0.2% Triton X-100 10 min. The sections were then incubated with universal blocking solution, followed by overnight incubation with primary antibodies: anti- F2r IgG from Santa Cruz Biotechnology (sc-13503) and anti-Ctsk IgG (Santa Cruz, sc-48353).

### Statistical analysis

Experimental data are reported as mean ± SEM of 3-5 independent samples in each group. Data were analyzed with the Student's t test or two-way analysis of variance using GraphPad Prism (GraphPad Software, Inc., USA) statistical program. Values were considered statistically significant at *p<0.05, **p<0.01, ***p<0.001. ns, no significant difference. Error bars depict SEM. Figures are representative of the data.

## Results

### F2r is highly expressed in osteoclasts

**Figure 1:**
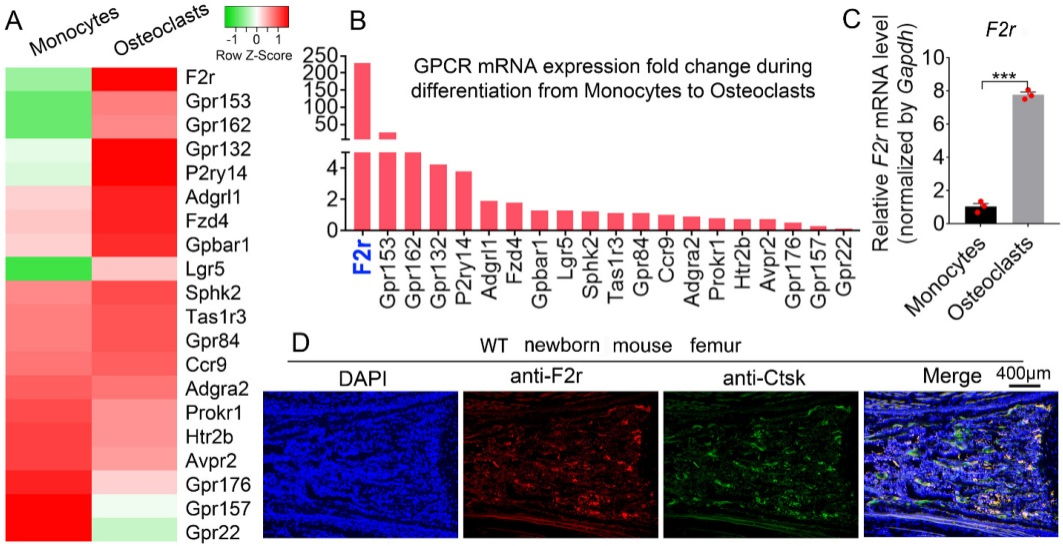
** F2r is highly expressed in osteoclasts.** (**A**) Heatmap for 20 GPCRs expression in wild type mouse bone marrow monocytes (MBMs) and osteoclasts induced by M-CSF (10 ng/ml) and RANKL (10 ng/ml) from RNA-seq analysis. (**B**) Fold change of GPCRs mRNA expression in monocytes and osteoclast from RNA-seq data. (**C**) qRT-PCR analysis of *F2r* mRNA expression in monocytes and osteoclasts by compared with *Gapdh*. One dot represents one sample (n=3). (**D**) Immunofluorescence (IF) stain of anti-F2r (red) and anti-Ctsk (green) on new born wild type (WT) mouse femur section. Results are presented as mean ± SEM of triplicate independent samples. ***p<0.001.

To explore the roles of GPCRs in osteoclastogenesis, we performed genome-wide expression analysis using WT murine bone marrow monocytes (MBMs) and osteoclasts induced by M-CSF and RANKL. Interestingly, our RNA-sequencing (RNA-seq) analysis data showed significantly upregulated expression levels of several G-proteins in osteoclasts, with F2r demonstrating the greatest increase in expression in osteoclasts compared to monocytes (**Fig. 1A, B**). This indicates that F2r may play an important role in osteoclasts. We then performed qRT-PCR to confirm the expression change in F2r between monocytes and osteoclasts induced by M-CSF and RANKL (**Fig. 1C**). Notably, we found that F2r was increased by 85% in osteoclasts at the mRNA level compared to monocytes (**Fig. 1C**). Additionally, F2r was widely expressed in osteoclasts of newborn mouse femur, as shown by immunofluorenscence (IF) data merged with that of the osteoclasts marker gene Ctsk (**Fig. 1D**). These data indicate that F2r may play an important role in osteoclasts.

### F2r knockdown promotes osteoclast formation and function

**Figure 2:**
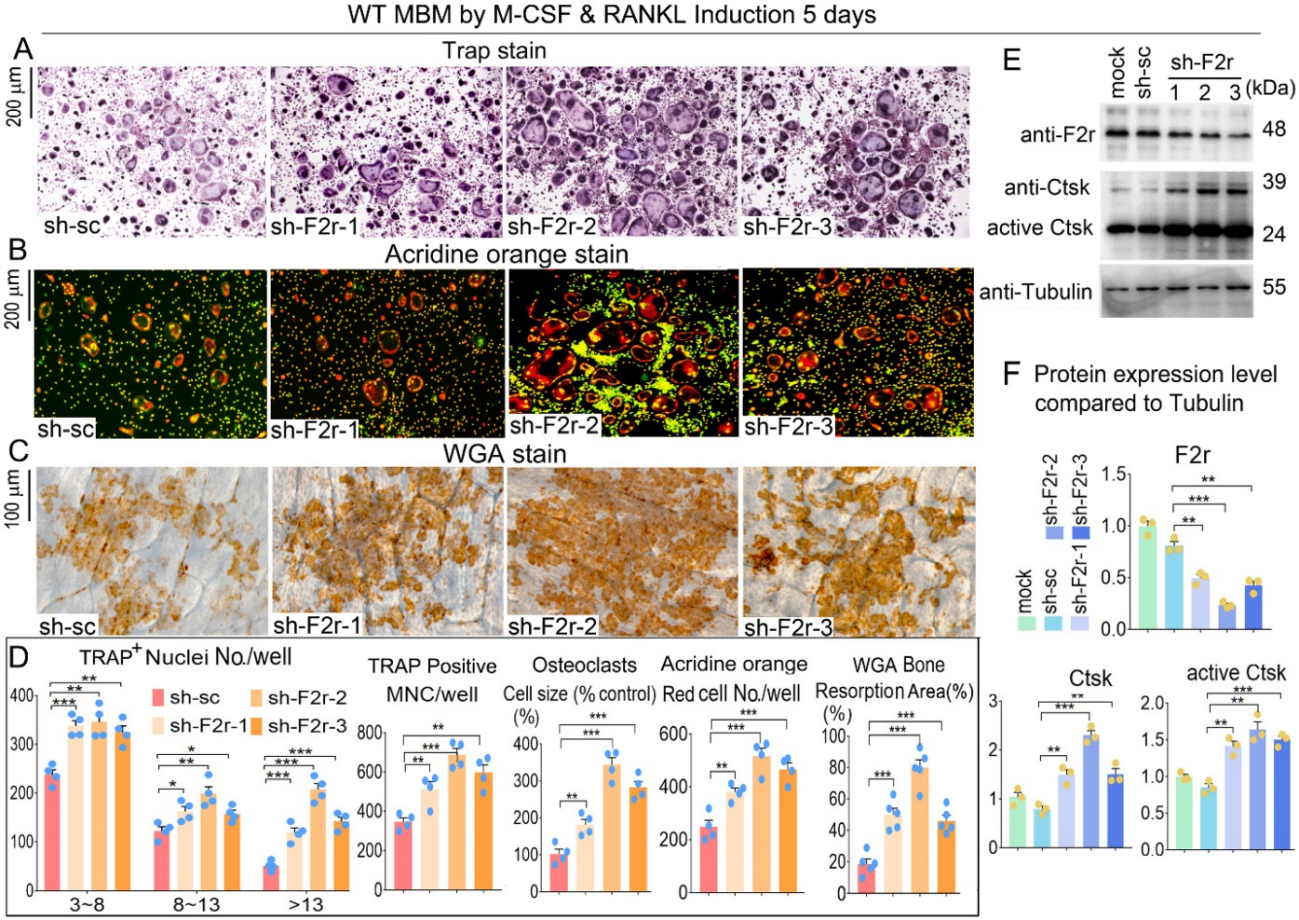
** F2r knockdown promotes osteoclastogenesis**. (**A**) TRAP stain and (**B**) Acridine orange (AO) stain of sh-sc and sh-F2r infected MBMs that induced 5 days by M-CSF and RANKL in 12-well-plate. (**C**) Wheat germ agglutinin (WGA) stain of osteoclasts on bone slices to detect bone resorption area. (**D**) Quantification data of **A-C**. TRAP-positive MNCs (multinucleated cells, ≥3 nuclei). (**E**) Western blot of F2r and Ctsk protein expression level in sh-sc and sh-F2r infected MBMs that induced 5 days by M-CSF and RANKL. (**F**) Quantification data of **E** by image J. Tubulin was used as a control. Protein expression level in the mock group was normalized as 1. Results are presented as mean ± SEM; one dot represents one sample. n≥3. **p<0.01, ***p<0.001.

To further investigate the role of F2r in osteoclasts, we silenced F2r expression by lentiviral-mediated expression of short hairpin RNA (using pLKO.1-shRNA-F2r) in MBM. Infected MBM was stimulated for 5 days by M-CSF and RANKL to generate osteoclasts. We then conducted osteoclast functional detection experiments to evaluate the effects of F2r silencing. F2r knockdown (sh-F2r) or control (sh-sc) lentivirus were transfected into osteoclast precursors with 5% pLKO.1 GFP shRNA, and GFP expression observed in mature osteoclasts (**Fig. S1**) indicated that successful lentiviral transfection into osteoclasts had occurred. TRAP stain data showed that F2r silencing drastically promoted osteoclasts activity and formation, and also drastically increased osteoclasts size, number and multinucleated cells (**Fig. 2A, D**). These results demonstrated that the knockdown of F2r positively regulates osteoclastogenesis. To detect the effect of F2r knockdown on osteoclast function, we performed Acridine orange (AO) stain on mature osteoclasts. Higher acidification levels were found in sh-F2r group by AO stain (**Fig. 2B, D**). To further study the role of F2r in osteoclast activity, we performed bone resorption assays and analyzed bone resorption area by wheat germ agglutinin (WGA) stain (**Fig. 2C, D**). The results demonstrated that the total resorption area in sh-F2r treated osteoclasts was significantly higher than that of control osteoclasts (**Fig. 2C, D**), indicating that F2r plays an important role in osteoclast function. Moreover, we analyzed actin ring formation, which showed that F2r knockdown increased F-actin ring formation, which is important during osteoclast maturation (**Fig. S2A, B**). In addition, the expression of osteoclast marker gene Ctsk was significantly upregulated (**Fig. S2B**). We detected the efficiency of F2r knockdown through western blot, and found that the F2r protein expression levels were significantly decreased in sh-F2r treated osteoclasts, and sh-F2r-2 showed a higher knockdown efficiency (**Fig. 2E, F**). Moreover, we found that F2r knockdown elevated the protein levels of osteoclast marker gene Ctsk, as well as the protein levels of activated Ctsk (**Fig. 2E, F**). Taken together, these data indicated that F2r knockdown promotes osteoclast formation and increases osteoclast-mediated bone resorption.

### F2r overexpression inhibited osteoclastogenesis

**Figure 3:**
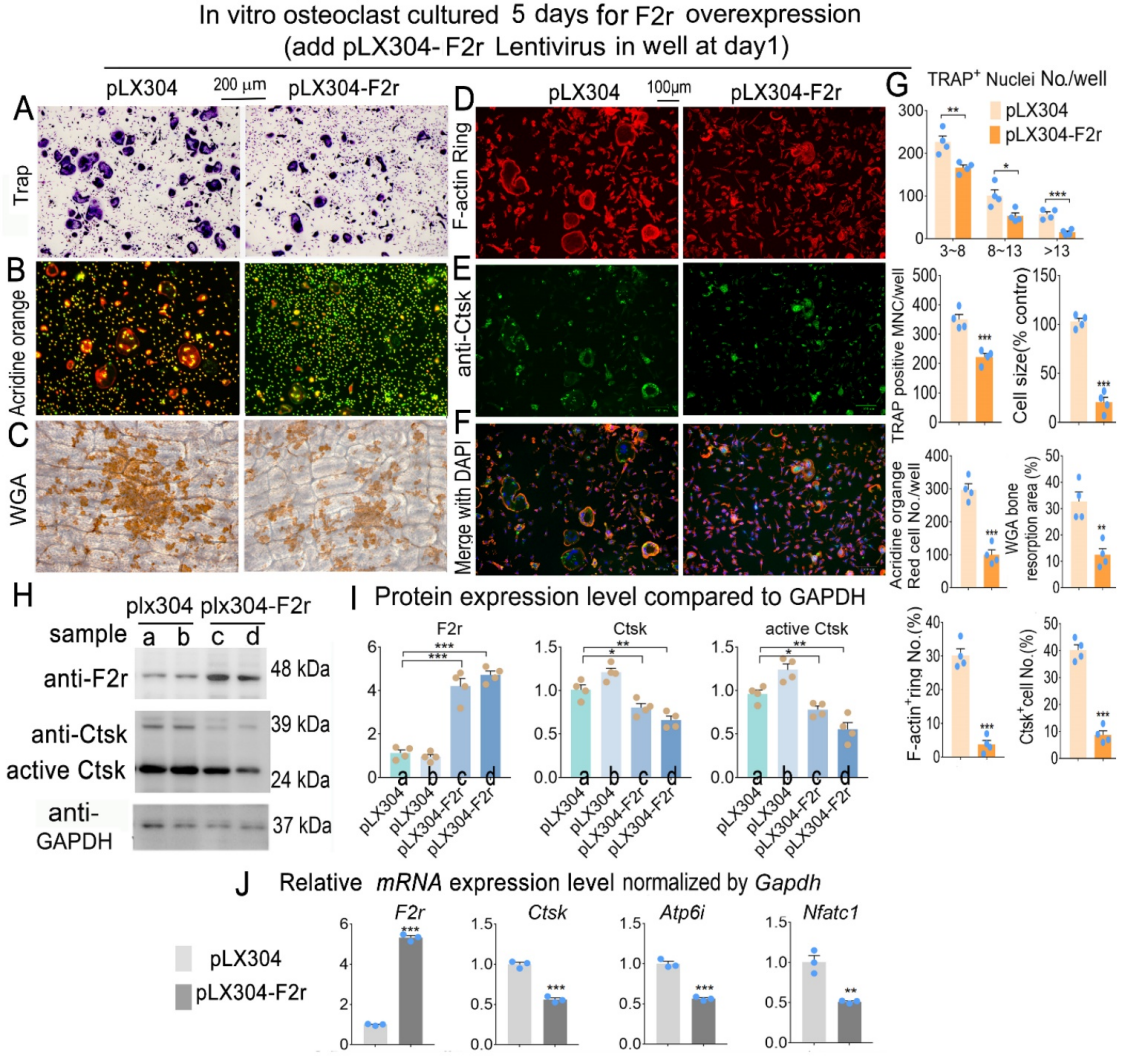
** F2r overexpression inhibited osteoclastogenesis.** (**A**) TRAP stain and (**B**) Acridine orange stain of pLX304 and pLX304-F2r infected MBMs that induced 5 days by M-CSF and RANKL. (**C**) Wheat germ agglutinin (WGA) stain analysis of osteoclast on bone slices to detect bone resorption area of induced MBMs. (**D**) Fluorescence microscopy of F-actin ring stain and (**E**) anti-Ctsk immunofluorescence (IF) stain in mature osteoclasts. (**F**) Overlap of D, E was detected as a yellow-orange area in the merged image. (**G**) Quantification data of A-F. (**H**) Western blot of F2r and Ctsk protein expression level in pLX304 (a,b) and pLX304-F2r (c,d) infected osteoclasts. GAPDH was used as a control. Protein expression level in pLX304 group normalized as 1. (**I**) Quantification data of H. (**J**) qRT-PCR was carried out to measure *F2r, Ctsk, Atp6i* and *Nfatc1* mRNA levels relative to *Gapdh* in osteoclasts. One dot represents one sample. Results are presented as mean ± SEM; n≥3. *p<0.05, **p<0.01, ***p<0.001.

To investigate whether F2r gain-of function could regulate osteoclastogenesis, we overexpressed F2r through lentiviral-mediated expression of pLX304-F2r in MBM. We performed Trap stain and found that F2r overexpression significantly inhibited the formation of osteoclasts, significantly decreased osteoclast size, and inhibited the formation of multi-nucleated osteoclasts (**Fig. 3A, G**). To further investigate the effects of F2r overexpression on osteoclast function, we performed Acridine orange (AO) stain in mature osteoclasts. Our data showed that extracellular acidification was significantly reduced by 66% in pLX304-F2r treated osteoclasts (**Fig. 3B, G**). Then, we performed wheat germ agglutinin (WGA) stain to observe the bone resorption area (**Fig. 3C**). Our data showed that the total bone resorption area of pLX304-F2r treated osteoclasts was 65% lower than that of control osteoclasts (**Fig. 3C, G**). In addition, we analyzed F-actin ring formation by IF stain, which showed that the formation of F-actin rings was severely impaired (85% reduction) in the F2r overexpression osteoclasts, along with regulation of the OC marker gene Ctsk (80% reduction) (**Fig. 3D-G**). Overall, these data indicated that F2r overexpression inhibits osteoclast formation and function. Moreover, our western blot data confirmed that F2r was successfully overexpressed by pLX304-F2r lentivirus infection in monocytes and osteoclasts (**Fig. 3H, I**). The analysis revealed that the protein levels of Ctsk and active-Ctsk were significantly decreased in the F2r overexpression group (pLX304-F2r) osteoclasts (**Fig. 3H, I**), while the expression of osteoclastogenesis marker genes *Ctsk, Atp6i* and *Nfatc1* were also significantly reduced in pLX304-F2r treated osteoclasts (**Fig. 3J**). Taken together, these results demonstrated that F2r is a key negative regulator of osteoclastogenesis.

### F2r inhibited osteoclast F-actin ring formation on bone slides

**Figure 4:**
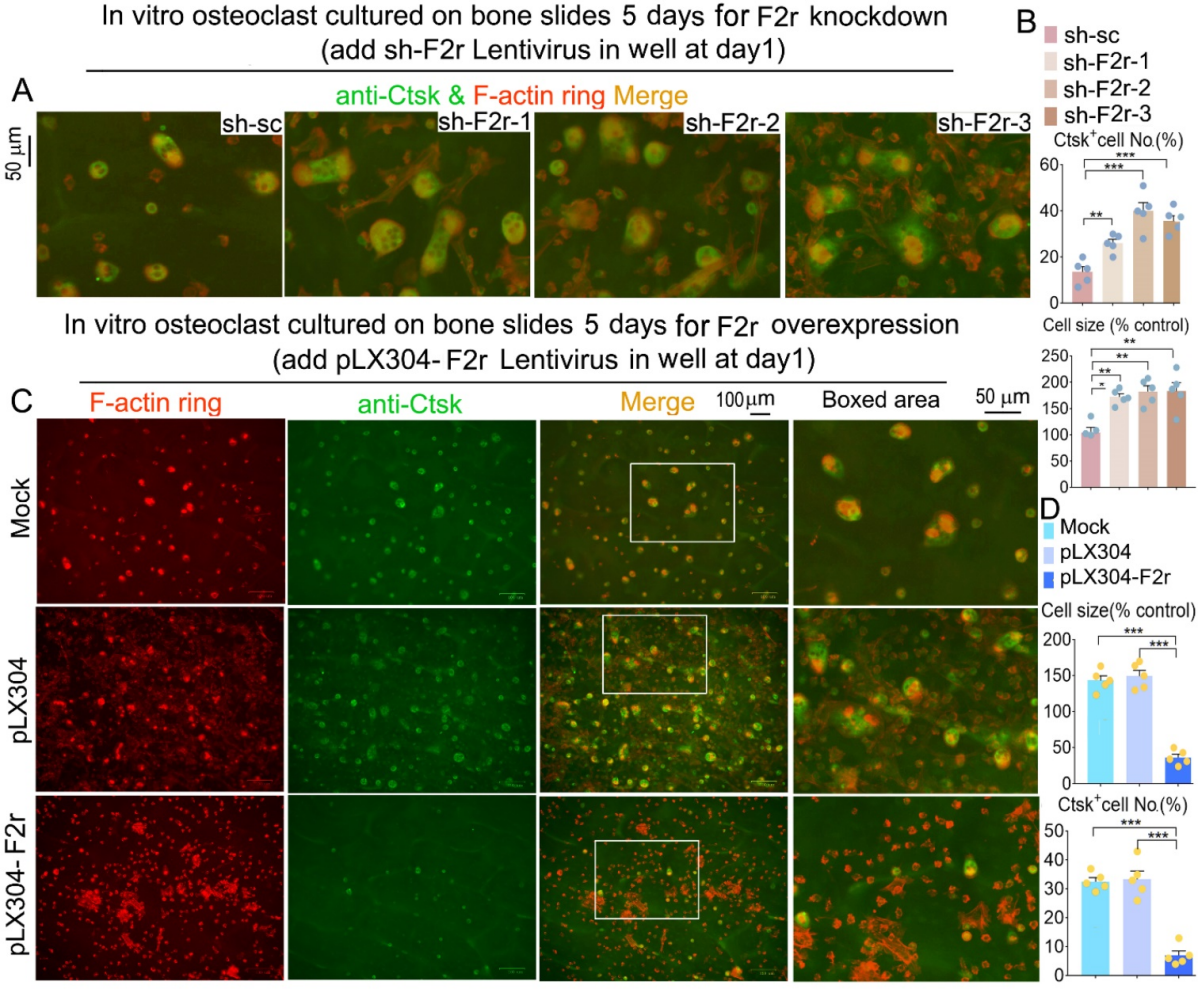
** F2r inhibited F-actin ring formation and Ctsk expression in mature osteoclasts on bone slides.** (**A**) Fluorescence microscopy of F-actin ring stain and anti-Ctsk immunofluorescence (IF) stain in mature osteoclasts from MBMs infected by sh-sc and sh-F2r on bone slides. Overlap detected as a yellow-orange area in the merged image. (**B**) Quantification data of **A**. (**C**) F-actin ring stain and anti-Ctsk IF stain for pLX304 and pLX304-F2r infected MBMs that were induced to mature osteoclasts on bone slides. (**D**) Quantification data of **C**. Results are presented as mean ± SEM; n≥4. *p<0.05, **p<0.01, ***p<0.001.

Next, we observed the formation of the F-actin ring, which is an important structure for osteoclast maturation 29. Equal numbers of MBM cells were seeded on each bone slice and induced by M-CSF and RANKL to generate osteoclasts. Our data revealed that F2r deficient osteoclasts exhibited a significant increase in F-actin ring formation, and the average size of the osteoclasts was also about 1.8 fold larger in these cells compared to control cells (**Fig. 4A, B and Fig S3**). We further examined the molecular distribution of Ctsk through IF staining. Ctsk expression in osteoclasts depleted of F2r showed a significant increase (**Fig. 4A, B and Fig S3**). Consistently, F-actin ring formation, and osteoclast size, and Ctsk expression were all significantly reduced in the F2r overexpression group (**Fig. 4C, D**). Thus, F2r may play a critical role in regulating Ctsk expression, resulting in a critical role of F2r in osteoclast-mediated bone resorption.

### F2r responds to RANKL induced osteoclastogenesis through regulating the Akt and NF-κB signaling pathways

**Figure 5:**
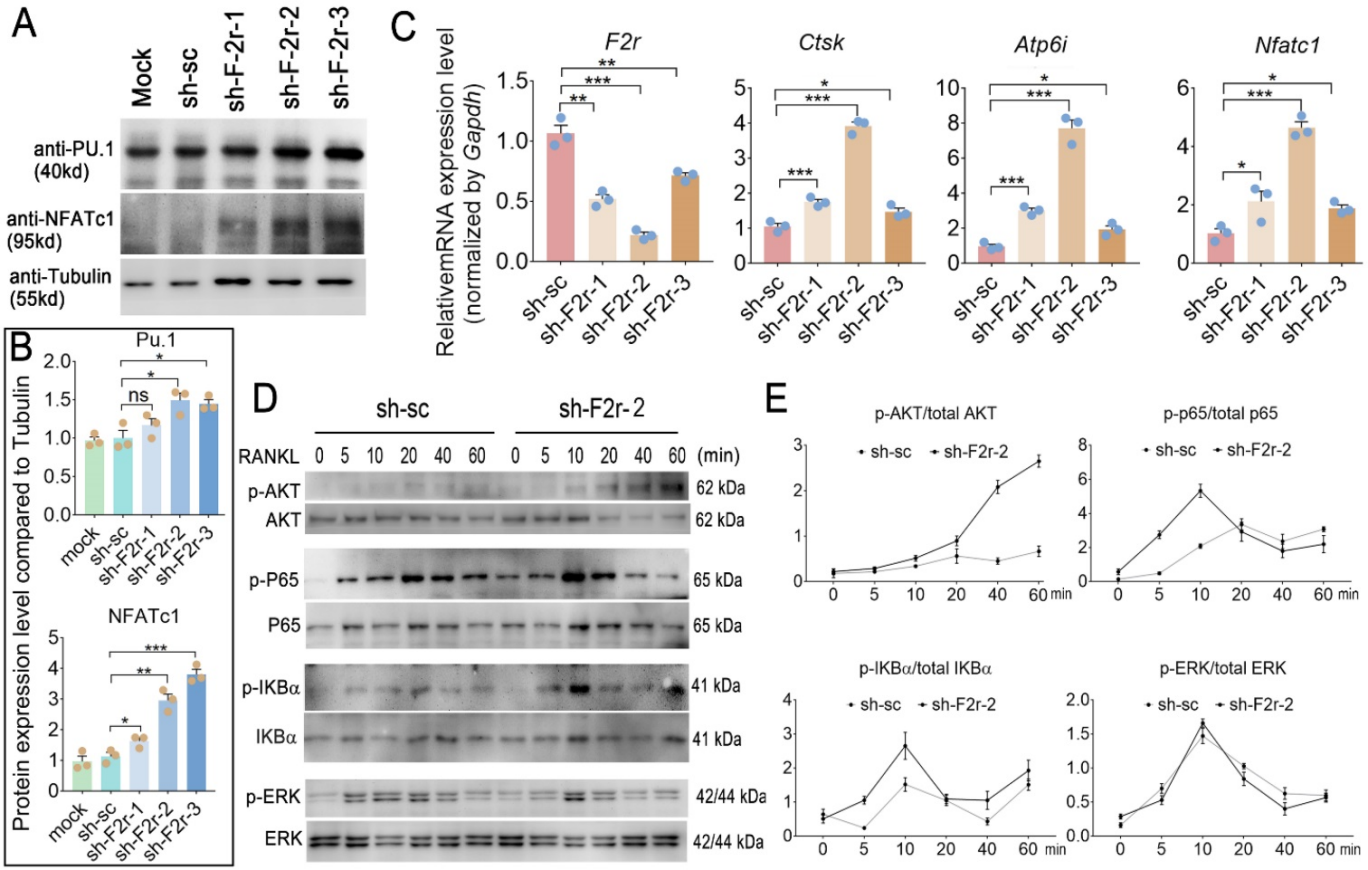
** F2r answered RANKL -induced MBMs through Akt and NFκB signaling pathways.** (**A**) Western blot analysis of Pu.1 and NFATc1 expression in osteoclasts at day 5 from MBMs infected by shRNA. Tubulin served as a control. (**B**) Quantification of A. Mock group protein expression normalized as 1. (**C**) RT-qPCR was used to detect *F2r, Ctsk, Atp6i* and* Nfatc1* mRNA levels relative to *Gapdh* in M-CSF and RANKL-induced MBMs. (**D**) Western blot analysis to detect Akt, p65, IKBα and Erk phosphorylation induced by RANKL in monocytes/macrophages infected sh-sc and sh-F2r-2. (**E**) Quantification of phospho-protein level versus total protein level by RANKL stimulation. One point represents one sample. Results are presented as mean ± SEM; n>3. *p<0.05, **p<0.01, ***p<0.001. ns, no significant difference.

We next sought to investigate the mechanism underlying how F2r negatively regulates osteoclastogenesis. In our western blot data, we found F2r deficiency in osteoclast enhanced transcription factor Pu.1 and NFATc1 expression, and NFATc1 showed more obvious changed (**Fig. 5A, B**). Interestingly, qRT-PCR analysis revealed that the mRNA expression levels of osteoclastogenesis marker genes *Ctsk, Atp6i* and *Nfatc1* were significantly increased in the F2r knockdown group (sh-F2r) in MBMs cultured with M-CSF and RANKL for 5 days (**Fig. 5C**). Further, to investigate the mechanism of F2r in osteoclast, we detected RANKL-induced signaling pathways related protein change in control cells and sh-F2r cells. sh-F2r and sh-sc transduced pre-osteoclast cells were starved for 3h and then stimulated with RANKL for 0-60 min (**Fig. 5D, E**). Western blot results showed p-Akt level significantly increased in sh-F2r infected cells (**Fig. 5D, E**). Moreover, RANKL-induced NFκB signaling pathway changed between sh-sc and sh-F2r knockdown group (**Fig. 5D, E**). Data showed that phosphorylation of p65 and IKBα level were significantly enhanced in early stage of RANKL stimulation in F2r knockdown cells compared with the control group (**Fig. 5D, E**). Besides, we also detected the levels of phosphorylated and non-phosphorylated Erk, and found no significant difference between the sh-sc and sh-F2r knockdown groups (**Fig. 5D, E**). These data suggested that F2r may attenuate osteoclastogenesis by inhibiting the Akt and NFκB signaling pathways.

## Discussion

**Figure 6:**
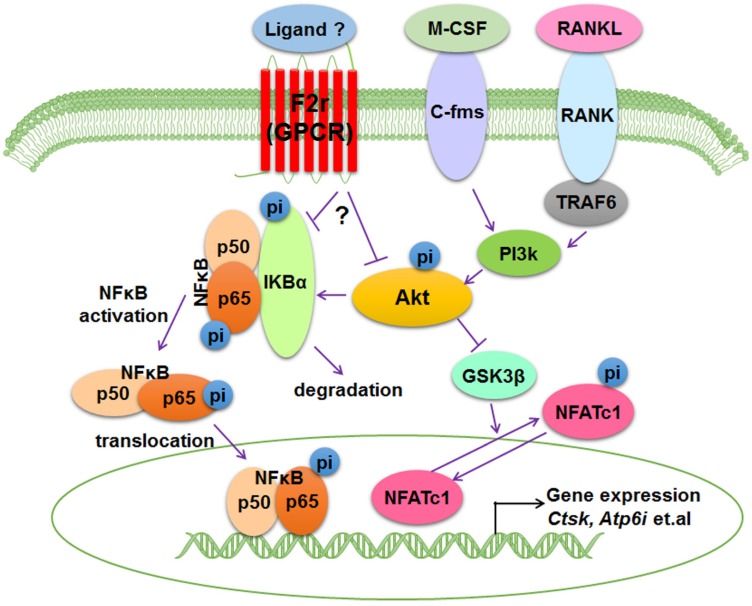
** Working model of F2r negative regulation of osteoclasts.** F2r negatively regulates osteoclastogenesis through inhibiting the F2r-Akt-GSK3β-NFATc1 and suppressing F2r-NFκB signaling pathways to regulate osteoclasts marker genes expression.

Bone is an important dynamic tissue whose remodeling is accomplished by the balance of osteoblasts and osteoclasts 35,36. Osteoclasts are terminally differentiated bone-resorbing cells that can remove mineralized bone matrix 37. In this study, we demonstrated that F2r is highly expressed in osteoclasts, and we proposed a model that F2r reduces osteoclast marker genes expression and antagonizes osteoclast formation by responding to RANKL stimulation to inhibit F2r-Akt-GSK3β-NFATc1 and F2r-Akt-NFκB signaling pathways (**Fig. 6**).

F2r, also known as PAR1, is a G-protein-coupled receptor (GPCR) which is activated by cleavage of the N-terminal domains by serine proteases 20,38,39. The PAR family also enables cells to respond to proteases in the extracellular environment in a nuanced and dynamic manner, such as plasmin, activated protein C, thrombocytin, PA-BJ, factor Xa, factor VIIa, kallikreins, cathepsin G, trypsin, matriptase and tryptase 40. Thrombin is the natural ligand of F2r 40,41. Currently, GPCRs remain a major target for bone related diseases drug discovery 38,39,42,43. F2r has been the object of large-scale drug development programs since the 1990s, and Vorapaxar and drotrecogin-α are approved F2r-targeted therapeutics 20. New understanding of the mechanisms underlying F2r's function and the discovery of improved strategies to modify F2r function has provided new opportunities for therapies targeting F2r 20. Previous reports have revealed that F2r plays a novel regulatory role in maintaining the hematopoietic stem cell pool in addition to the balance between hematopoiesis and bone structure 44, indicating that F2r plays a pivotal role in osteoclast and osteoblast mediated bone remodeling and bone homeostasis.

In our study, through RNA-seq and qRT-PCR we revealed that F2r expression significantly increased in osteoclasts after RANKL and M-CSF stimulation, suggesting that F2r may play an important role in osteoclasts. Recently, Jastrzebski et. al. demonstrated that F2r deletion causes enhanced osteoclastogenesis 45. However, the functions of F2r for osteoclast acidification, differentiation, activation, and bone resorption are still unknown; and without detailed mechanism studies, the mechanisms underlying the role of F2r in osteoclastogenesis remains unclear. In this study, we comprehensively investigated the roles of F2r in osteoclasts by loss-of function, gain-of function in primary cells. We used multiple lentivirus strains to sufficiently examine that F2r knockdown stimulated osteoclast marker genes expression, osteoclast differentiation, osteoclast formation, as well as triggered a significant increase in osteoclasts number, size, and activity. This in turn accelerated osteoclast maturation and acidification, and increased osteoclast-mediated bone resorption function, which indicates that F2r is a negative regulator for osteoclastogenesis. Moreover, F2r gain-of function blocked osteoclast acidification, formation, and maturation, which suggests that F2r plays a negative regulatory role in osteoclast maturation and bone resorption.

RANKL is pivotal receptor activator of the NF-κB ligand, and is also a critical cytokine required for osteoclast precursor cells survival, expansion, formation, and differentiation *in vitro* 46. Binding of RANKL to RANK is a pivotal step in osteoclast development, which causes activation of downstream critical signaling such as Akt and NF-κB 47-49. Here, we found that F2r limits Akt phosphorylation levels under RANKL stimulation, and F2r knockdown regulates transcription factor NFATc1 in osteoclasts, which indicates that activation of NFATc1 is responsible for the facilitation of RANKL-induced osteoclast formation. It has previously been shown that Akt induces osteoclast differentiation through inhibiting GSK3β signaling cascade, which can regulate NFATc1 translocation from the cytoplasm into the nucleus 5,48. Our results demonstrate that F2r knockdown cells exhibited significantly increased pAkt levels, as well as significantly enhanced phosphorylation of p65 and IKBα in early stages of RANKL stimulation, indicating that F2r may inhibit osteoclast differentiation through suppressing the Akt-GSK3β-NFATc1 signaling cascade. However, how F2r activates Akt is still unclear and warrants further investigation. Interestingly, Jastrzebski et.al. detected the p65 expression in cytoplasmic and nuclear under RANKL (30 ng/ml) stimulated 15 min, and found no significant differences between control and F2r-defcienct cell 45. In our study, F2r knockdown increased p65 and IKBα phosphorylation level under RANKL (10 ng/ml) stimulated at 5 min and 10 min, indicating that F2r responds to RANKL signaling at early stages of osteoclastogenesis. It could due to the dosing and treatment timing was different from the current study. Moreover, Akt phosphorylation promotes NF-κB activation that can translocate to the nucleus regulate different genes expression and influence osteoclast growth and fusion 50-52. In osteoclasts, it needs further investigation which ligands activate F2r to inhibit osteoclast formation. Here, our data showed that F2r as a transmembrane protein that responses the stimulation of RANKL to inhibit phosphorylation level of Akt and activation of NF-κB signaling. These data demonstrated that F2r can respond to RANKL stimulation via inhibiting the NF-κB signaling pathway at the early stages of osteoclastogenesis, and through limiting Akt-GSK3β-NFATc1 signaling pathway which lead to a reduction in the expression of osteoclast marker genes. Erk signaling is also involved in osteoclast formation, proliferation, and differentiation 50,53. However, we found no significant difference in the p-Erk level between sh-sc and sh-F2r treated groups under RANKL stimulation, indicating that F2r may not regulate Erk signaling pathway under RANKL stimulation during osteoclast proliferation and differentiation. Notably, the differential levels of F2r silencing or overexpression were consistent with the respective signaling pathway activation or inhibition seen in the osteoclasts with F2r silencing or overexpression, which indicates that targeting F2r through shRNA may be an effective target for bone disease. In summary, our results from both loss-of-function and gain-of-function strategies revealed that F2r is a key negative regulator of osteoclastogenesis, which inhibits the Akt-GSK3β-NFATc1 and NFκB signaling pathways, suggesting that F2r can be as a novel therapeutic target for bone diseases, such as osteoporosis.

## Supplementary Material

Supplementary figures and tables.

## Abbreviations

OCs: osteoclasts
MBMs: mouse bone marrow monocytes
GPCRs: G-protein-coupled receptors
Ctsk: cathepsin K
M-CSF: macrophage colony-stimulating factor
RANKL: receptor activator of NF-κB ligand
NFATc1: nuclear factor of activated T-cells, C1
TRAP: tartrate-resistant acid phosphatase 5
