# Supplementary material for: F2r negatively regulates osteoclastogenesis through inhibiting the Akt and NFκB signaling pathways
Source: Int J Biol Sci. 2020 Mar 12;16(9):1629–39. doi: 10.7150/ijbs.41867 (PMC7097923; doi:10.7150/ijbs.41867)
Supplement: Supplementary file 1 — Supplementary figures and tables. [file ijbsv16p1629s1.pdf]

F2r negatively regulates osteoclastogenesis through inhibiting the Akt and NFκB signaling pathways

Yan Zhang[1,2], He Wang[2], Guochun Zhu[2], Airong Qian[1]*, Wei Chen [2]*

[1]Laboratory for Bone Metabolism, Key Lab for Space Biosciences and Biotechnology, School of Life Sciences, Northwestern Polytechnical University, Xi'an, Shaanxi, 710072, China.

[2]Department of Pathology, The School of Medicine, University of Alabama at Birmingham, Birmingham, AL 35294, USA.

Supplemental Materials

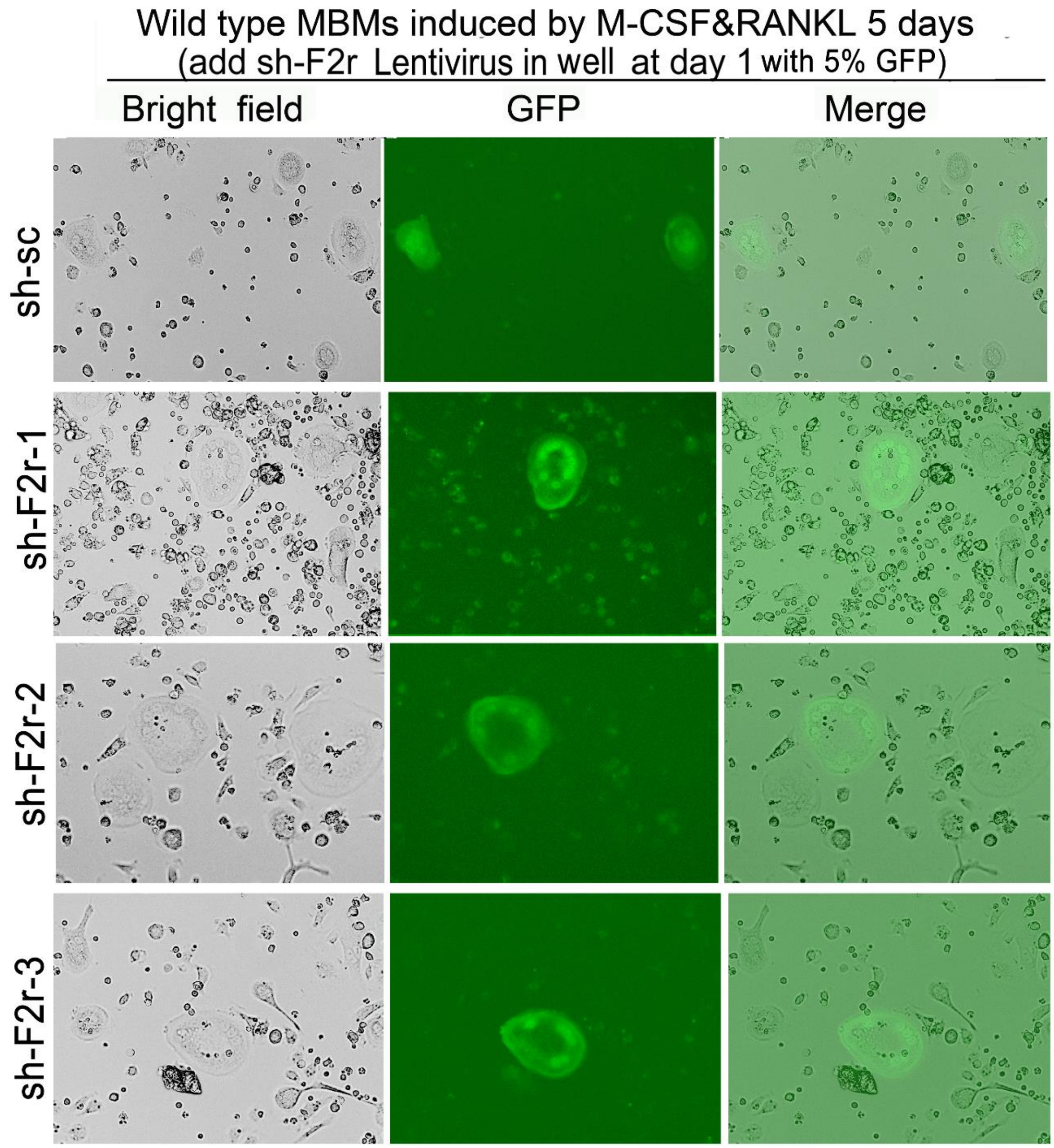

Supplemental Fig. 1. sh-sc and sh-F2r were successfully transfected into osteoclasts with GFP. F2r knockdown (sh-F2r) or control (sh-sc) lentivirus was transfected into osteoclast precursors with 5% GFP (pLKO.1 GFP shRNA) lentivirus, and GFP expression was observed in mature osteoclasts.

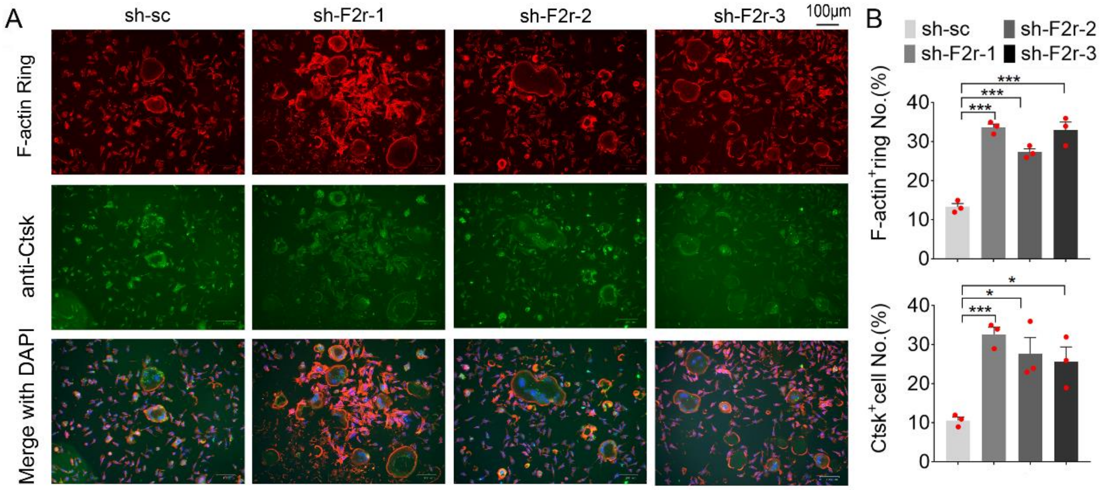

Supplemental Fig. 2 Loss of F2r promotes osteoclast formation. (**A**) Fluorescence microscopy of F-actin ring stain and anti-Ctsk IF stain in mature osteoclasts that from MBMs infected sh-sc and sh-F2r lentivirus. Overlap detected as a yellow-orange area in the merged image. (**B**) Quantification data of A. One point represents one well. Results are presented as mean ± SEM; n=3. **p*<0.05, ****p*<0.005.

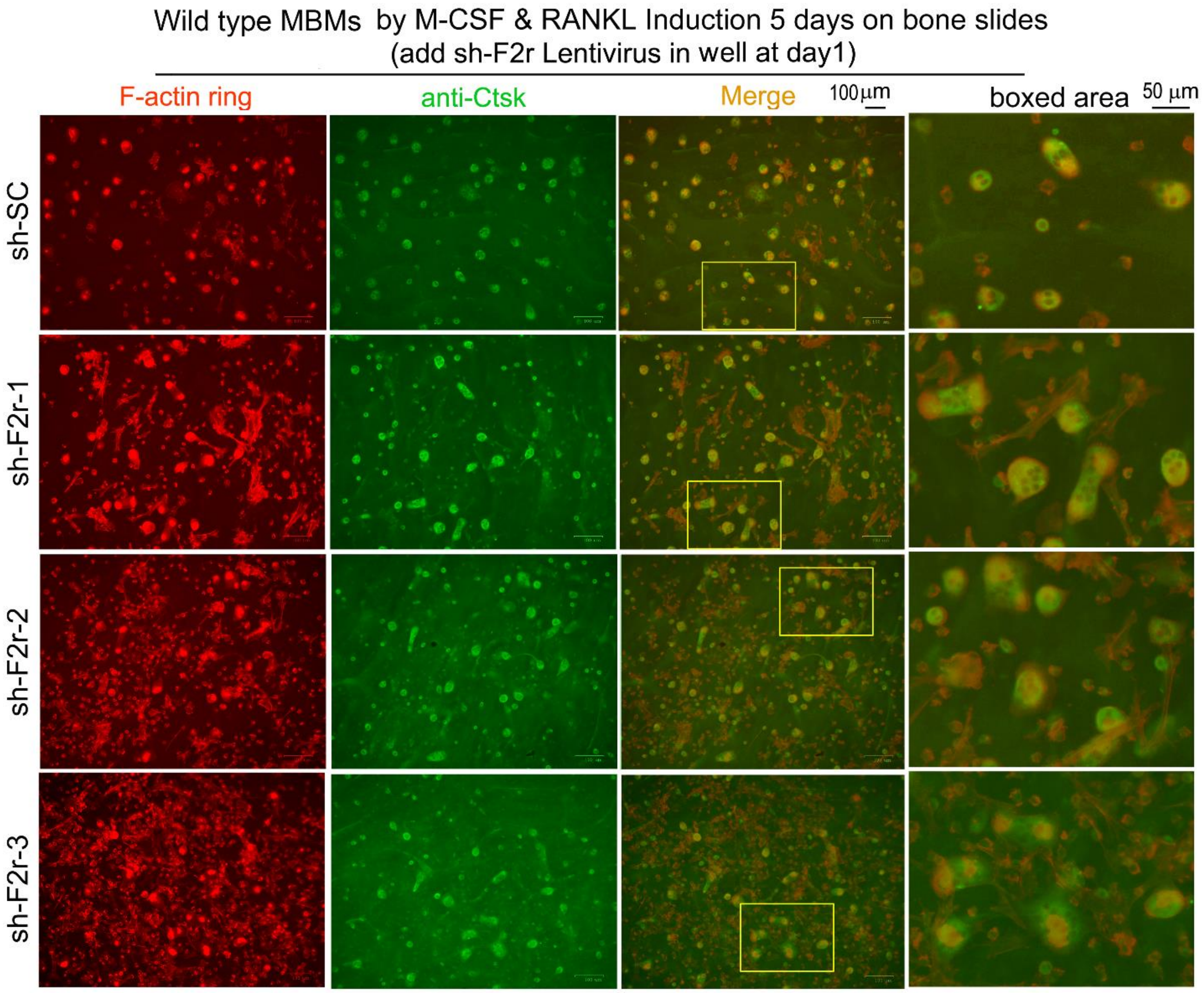

Supplemental Fig. 3 F2r knockdown promoted F-actin ring formation and Ctsk expression in mature osteoclast on bone slides. Fluorescence microscopy of F-actin ring stain and anti-Ctsk immunofluorescence (IF) stain in mature osteoclast that from MBMs infected by sh-sc and sh-F2r on bone slides. shRNA lentivirus were added on day 1 by M-CSF and RANKL induced, then cells were performed stain at day 5. Overlap detected as a yellow-orange area in the merged image.

Supplemental Table 1. Primers used for qRT-PCR

Gene symbol	qRT-PCR Primer sequences (5'-3')
F2r	Forward: 5'- TGAACCCCCGCTCATTCTTTC -3'
	Reverse: 5'-CCAGCAGGACGCTTTCATTTTT-3'
Ctsk	Forward: 5'-GGGCTCAAGGTTCTGCTGC-3'
	Reverse: 5'-TGGGTGTCCAGCATTTCCTC-3'
Nfatc1	Forward: 5'-TGCCTTTTGCGAGCAGTATCT-3'
	Reverse: 5'-CAGGCAAGGATGGGCTCATAT-3'
Atp6i	Forward: 5'-CACAGGGTCTGCTTACAACTG-3'
	Reverse: 5'-CGTCTACCACGAAGCGTCTC-3'
Gapdh	Forward: 5'-GACCACAGTCCATGCCATCAC-3'
	Reverse: 5'-TCCAC CACCCTGTTGCTGTAG-3'
